# Nocturnal blood pressure: pathophysiology, measurement and clinical implications. Position paper of the European Society of Hypertension

**DOI:** 10.1097/HJH.0000000000004053

**Published:** 2025-06-12

**Authors:** Gianfranco Parati, Martino F. Pengo, Alberto Avolio, Michel Azizi, Tomas Lucca Bothe, Michel Burnier, Francesco Paolo Cappuccio, Alejandro De La Sierra, Cristiano Fava, Mariela M. Gironacci, Satoshi Hoshide, Kazuomi Kario, Anastasios Kollias, Carolina Lombardi, Giuseppe Maiolino, Simona Maule, Krzysztof Narkiewicz, Takayoshi Ohkubo, Paolo Palatini, Jean Luis Pepin, Pantelis Sarafidis, Aletta Elisabeth Schutte, Alessandro Silvani, George Stergiou, Paolo Verdecchia, Giuseppe Mancia, Grzegorz Bilo

**Affiliations:** a Istituto Auxologico Italiano, IRCCS, Department of Cardiology, Ospedale San Luca; b Department of Medicine and Surgery, University of Milano-Bicocca, Milan, Italy; c Macquarie Medical School, Faculty of Medicine, Health and Human Sciences, Macquarie University, Sydney, Australia; d Université Paris Cité, INSERM, CIC1418, AP-HP, Hôpital Européen Georges-Pompidou, Hypertension Department and DMU CARTE, Paris, France; e Charité – Universitätsmedizin Berlin, Institute of Physiology, Center for Space Medicine and Extreme Environments, Berlin, Germany; f University of Sydney, Sydney School of Health Sciences, Sydney, Australia; g Faculty of Biology and Medicine, University of Lausanne, Lausanne, Switzerland; h University of Warwick, Warwick Medical School, and University Hospital Coventry & Warwickshire NHS Trust, Coventry, UK; i Hypertension Unit. Department of Internal Medicine. Hospital Mutua Terrassa. University of Barcelona, Terrassa, Spain; j Department of Medicine, University of Verona, Verona, Italy; k Universidad de Buenos Aires, Facultad de Farmacia y Bioquímica, IQUIFIB (UBA-CONICET), Buenos Aires, Argentina; l Division of Cardiovascular Medicine, Jichi Medical University School of Medicine, Shimotsuke, Japan; m Hypertension Center STRIDE-7, National and Kapodistrian University of Athens, School of Medicine, Third Department of Medicine, Sotiria Hospital, Athens, Greece; n Clinica Medica 3, Department of Medicine, Azienda Ospedale Università Padova, Padova, Italy; o Autonomic Unit and Hypertension Unit, Department of Medical Sciences, University of Turin, Turin, Italy; p Department of Hypertension and Diabetology, Medical University of Gdansk, Gdansk, Poland; q Tohoku Institute for Management of Blood Pressure, Sendai, Japan; r Studium Patavinum, Department of Medicine. University of Padova, Padua, Italy; s University Grenoble Alpes, HP2 Laboratory, Inserm U-1300, CHU Grenoble Alpes, Grenoble, France; t 1st Department of Nephrology, Aristotle University of Thessaloniki, Hippokration Hospital, Thessaloniki, Greece; u School of Population Health, University of New South Wales; The George Institute for Global Health, Sydney, Australia; v Department of Biomedical and Neuromotor Sciences, University of Bologna, Bologna; w Associazione Umbra Cuore e Ipertensione-OTS and Department of Cardiology, Hospital S. Maria della Misericordia, Perugia, Italy

**Keywords:** blood pressure monitoring, chronotherapy, circadian rhythms, nocturnal blood pressure, nondipping

## Abstract

Interest in the pathophysiology, measurement, and clinical implications of nocturnal blood pressure (BP) has significantly increased due to its strong association with cardiovascular risk, and its importance was recognized by the 2023 European Society of Hypertension (ESH) guidelines. Nocturnal BP regulation is complex and multifactorial, involving sleep-wake cycle, circadian rhythms, autonomic nervous system, renin-angiotensin-aldosterone system, and renal mechanisms.

24-h ambulatory blood pressure monitoring is currently the reference method for nocturnal BP assessment. Home BP monitoring, with specially designed, validated devices with nocturnal BP measuring function, may also be used, while new cuffless and wearable technologies hold great potential but require further validation.

Nocturnal BP phenotypes of clinical interest include nocturnal hypertension, increased nocturnal BP variability and altered day-night BP fluctuations. Among those, isolated nocturnal hypertension may be considered a type of masked hypertension. BP variability has prognostic relevance, as do the day-night BP changes, i.e. the nocturnal BP “dipping”.

Nocturnal hypertension and nondipping are particularly prevalent in individuals with autonomic neuropathies, sleep disorders (e.g., obstructive sleep apnoea), kidney disease, and metabolic or endocrine disorders, and are linked to hypertension mediated organ damage and cardiovascular risk.

Therapeutic strategies targeting nocturnal BP remain debated. Chronotherapy (evening dosing of antihypertensives) has shown inconsistent results in clinical trials. Renal denervation and treatment of sleep-related breathing disorders may lower nocturnal BP and improve sleep quality. More research is needed to further clarify pathophysiology, measurement, therapeutic interventions, and overall management of nocturnal hypertension, issues on which this ESH position paper offers an in-depth review.

## INTRODUCTION

The interest on night-time blood pressure (BP) has increased progressively over time for several reasons. First, because night-time sleep amounts to about one third of people's life, proper attention to its cardiovascular physiology and pathophysiology is obvious. Moreover, during sleep there is a profound rearrangement of the mechanisms involved in cardiovascular regulation, including those operating via the autonomic nervous system, the endocrine system and the kidney. This rearrangement may profoundly affect the pathophysiological factors operating in cardiovascular disease. Finally, night-time BP is a well established predictor of cardiovascular events, possibly with a greater significance than daytime BP.

In this paper a number of important aspects of night-time BP will be addressed, thanks to the contribution of international acknowledged experts in this field, including physiological and pathophysiological issues, current methods of nocturnal BP assessment; and clinical implications of night-time BP, including its impact on hypertension diagnosis, prognostic significance, relevance in specific clinical settings and therapeutic aspects.

## PHYSIOLOGY AND PATHOPHYSIOLOGY OF NOCTURNAL BP

### Role of sleep and circadian rhythm in determining night-time BP

#### Evidence from animal studies

Studies in unrestrained cats have shown many years ago that sleep (in particular deep sleep) is accompanied by a BP reduction as well as by other BP alterations compared to the day time [1,2]. To summarize, systolic and diastolic BP fall progressively as slow wave sleep deepens and a further BP reduction can be observed during the rapid eye movement (REM) sleep phase, which is further characterized by short term BP variations compared to rather stable BP in the slow-wave sleep phases. This BP decrease is associated with a reduction in heart rate (due to both increase in vagal tone and reduction in sympathetic tone), a relatively small decrease in cardiac output and a large reduction of systemic vascular resistance, with inclusion of visceral, renal and skeletal muscle districts. Such systemic vasodilatation is largely due to reduction of sympathetic tone with an origin in the central nervous system because of its marked decrease in surgically sympathectomized cats; non neural mechanisms are also involved, however, because after sympathectomy the reduction of cardiac output persists, possibly because of a decreased venous return due to muscle relaxation. Furthermore, in the kidney the sleep-related vasodilatation is maintained after denervation probably due to the need of blood flow preservation via autoregulation; and finally, the arterial baroreflex plays an important buffering role because after sino-aortic denervation the REM-sleep dependent BP fluctuations show a striking potentiation, sometimes with BP values below the level of brain perfusion. The BP reduction is limited also via arterial chemoreceptor stimulation.

Further questions have been addressed more recently, one of them being how much is the participation of the circadian BP rhythm to the nocturnal BP fall. Under unmasking conditions of forced desynchrony, the circadian rhythm of BP reveals a peak in the late subjective day. However, the peak-to-trough amplitude of this rhythm is approximately 4 mmHg [3], significantly lower than the 10–20 mmHg characterizing the typical nocturnal BP reduction [4]. This comparison suggests that the direct contribution of the circadian rhythm to the nocturnal BP decline is limited.

Nevertheless, the circadian rhythm strongly influences the rhythms of wakefulness and sleep. Sleep and wakefulness, in turn, modulate sympathetic and parasympathetic activity to resistance vessels and the heart. Additionally, wakefulness involves changes in posture, leading to alterations in venous return and effective circulating volume. Various factors such as food and water intake, metabolic rate due to physical exercise, and changes in energy availability to peripheral cells are also rhythmically influenced by wakefulness. These changes help entrain peripheral molecular clocks [5,6]. This suggests that the physiological changes induced by the sleep-wake cycle may also mask the BP fluctuations directly controlled by intrinsic circadian mechanisms, resulting in their reinforcement in physiological conditions or in their weakening in conditions of circadian misalignment. The occurrence of inappropriate cardiovascular activity at night in the face of local circadian clocks promoting rest and repair may contribute to explain the increased cardiovascular risk associated with high nocturnal BP [7].

Research on animal models and in humans supports the conclusion that decreases in cardiac output and increases in vascular resistance occur during the daily rest period [8,9] The rest period, or dark period in human subjects, is associated with a decrease in heart rate, attributed to the circadian rhythm's effect through the autonomic nervous system. The consequent decrease in cardiac output is partly compensated by increased peripheral vascular resistance, influenced by local blood flow autoregulation and circadian clocks.

Changes in BP and heart rate between sleep and wakefulness are indeed largely due to modulation of the autonomic nervous system. In particular, sympathetic activity to blood vessels mainly drives the changes in arterial pressure between non-REM sleep, wakefulness, and REM sleep [10]. The role of humoral factors was also identified in this regard: sleep-related fall in arterial BP is weaker in mouse models lacking orexin peptides, demonstrating an important direct or indirect role for these peptides in BP regulation during sleep [11], which translates to human subjects [12].

The overlap of brain structures responsible for autonomic control and wakefulness-sleep rhythms is evident in the brainstem and hypothalamus. Bidirectional projections between these structures establish a positive feedback loop between the baroreceptor reflex and arousal [13,14] and may help coordinate transitions between different brain states and autonomic activities.

In conclusion, the interplay between the sleep-wake cycle and intrinsic circadian rhythms in optimal BP regulation involves mechanisms operating at different timescales. The circadian rhythm operates over hours, sleep architecture over minutes, and sleep microstructure over seconds. While the direct contribution of the circadian rhythm to the nocturnal BP decline may be limited, the sleep-wake cycle serves as a powerful indirect masking mechanism for circadian BP control. Understanding these intricate relationships requires consideration of multiple factors across different temporal scales.

#### Evidence from human studies

In 1983, through the use of 24 h intra-arterial continuous ambulatory BP monitoring provided by the Oxford method [15], it was possible to precisely characterize the occurrence of night-time BP decrease in humans in daily life conditions, suggesting modulation of BP variability following the circadian rhythm [16]. The authors concluded that changes in BP are closely linked to sleep itself, irrespective of whether it occurs during the day or night. This emphasizes the coexistent influence of sleep and the circadian rhythm, as discussed in the previous section.

Focusing on sleep, a two-process model has been proposed that incorporates the circadian process, a 24-h internal clock modulating various functions, and the homeostatic model, representing sleep pressure. These models, regulated by genetic variations and aging, govern the sleep-wake cycle and impact brain function [17].

The interaction of circadian rhythms, sleep architecture, and sleep microstructure plays a crucial role in night-time BP variations. Non-REM sleep, characterized by predominant parasympathetic tone, correlates with lower values of heart rate and BP. Conversely, REM sleep exhibits high BP variability resembling wakefulness, accompanied by an increase in sympathetic activity to the skeletal muscles. Melatonin, a hormone regulated by the circadian rhythm and light exposure, can also affect cardiovascular regulation. Studies suggest melatonin's potential to increase nitric oxide and decrease norepinephrine, enhancing vasodilation [18]. Lower melatonin levels in hypertensive individuals are associated with higher nocturnal BP.

Sleep microstructure characteristics, including arousals and cyclic alternating pattern and sleep-related movement or breathing disorders (e.g. leg movements and/or sleep apnoea), further influence BP regulation at night. Sleep duration also plays a role, with sleep deprivation leading to elevated night-time BP. Isolated nocturnal hypertension appears more related to environmental factors than genetic markers, emphasizing the importance of sleep duration. Conversely, extending sleep duration in short sleepers induces changes in 24-h BP profiles [19] (Table 1).

**TABLE 1:** Key insights from animal and human studies regarding the role of sleep and circadian rhythms in BP regulation

Aspect	Animal studies	Human studies
Nocturnal BP regulation	Interplay of central and peripheral circadian clocks, fluid balance, sleep-wake behaviour, and autonomic nervous system control.	Limited direct contribution of circadian rhythm. Sleep is a powerful masking factor.
Role of autonomic nervous system	The modulation of sympathetic activity to blood vessels is key to BP control during sleep. Bidirectional interaction between the arterial baroreflex and arousal.	
Influence of melatonin	Melatonin potentially affects cardiovascular regulation	Lower melatonin levels associated with higher nocturnal BP
Impact of sleep structure	Sleep micro and macrostructure both influence BP regulation at night	
Environmental vs. genetic factors	Environmental and genetic factors both contribute to BP control during sleep	
Hypoxia effects on nocturnal BP	Intermittent hypoxia elevates BP, with a prominent role played by the arterial chemoreflex	

Hypoxia, modelled in healthy subjects through intermittent hypoxia exposure, leads to increased night-time BP [20]. Prolonged hypoxia, as observed in high-altitude exposure, demonstrates similar nocturnal BP elevations [21]. These findings were obtained through 24 h ambulatory blood pressure monitoring (ABPM), an approach which is known however to suffer from a series of limitations, Including disrupted sleep due to cuff inflation. Alternative solutions for nocturnal BP monitoring are currently being considered, such as nocturnal home BP measurement or ideally the future availability of validated cuffless BP monitoring devices which are currently under investigation (see ad hoc paragraph).

Physiological mechanisms regulating nocturnal BP are manifold and complex. Consequently, the nocturnal hypertension or nondipping pattern (i.e., weakening or lack of the physiological nocturnal BP fall) may be a common manifestation of very different conditions. Below, the most relevant mechanisms underlying nocturnal BP phenotype are discussed (Box 1).

Box 1 **Blood pressure control mechanisms and their influence on nocturnal hypertension and nondipping pattern**

**Table:** 

**Sodium and water handling** - Renal parameters like glomerular filtration rate (GFR) and sodium/water excretion exhibit circadian fluctuations - Hormones like aldosterone and vasopressin exhibit circadian rhythms influenced by the sleep-wake cycle. - Poor sodium excretion and impaired kidney function can lead to elevated night-time BP **Renin-Angiotensin System (RAS)** - Altered RAS function – specifically intrarenal RAS – correlates with nocturnal hypertension, especially in individuals with kidney diseases. - Alterations in circulating angiotensins levels contribute to isolated nocturnal hypertension. **Autonomic Nervous System (ANS)** - Autonomic nervous system activity changes importantly between wakefulness and sleep and between different sleep stages - Elevated sympathetic nervous activity is linked to nocturnal hypertension and the nondipping BP profile

### Sodium and water handling

There is a bidirectional relationship between renal function and nocturnal BP. Renal function is influenced by the circadian BP rhythm. Changes in glomerular filtration rate (GFR) and electrolyte and water excretion can vary significantly between daytime and night-time. Thus, these parameters should be measured separately during the day and night rather than relying on 24-h urine collections [22].

Furthermore, data from a study where renin, aldosterone, and cortisol levels were measured every 10 min over a 24-h cycle revealed that the 24-h aldosterone rhythm, generally thought to be purely circadian, is also influenced by the sleep-wake cycle, with aldosterone pulses being mainly related to plasma renin oscillations during sleep periods, whereas sleep, rather than an intrinsic circadian rhythm, is the main driver of fluctuations in these hormone levels [23,24]. Many complex factors may affect cyclic variations in such parameters, including sleep duration, season, temperature, light, meals, physical activity, body position and even microbiome variations with higher concentrations during night-time, however, of minor extent [25].

The circadian BP changes are influenced by sodium excretion and renal function as seen, for example, in chronic kidney disease (CKD), renal transplantation, pheochromocytoma or Cushing syndrome. In fact, studies suggest that poor sodium excretion and kidney function may contribute to elevated night-time BP and nondipping pattern and conversely, sodium restriction as well as diuretics and aldosterone antagonists can decrease nocturnal BP and possibly restore nocturnal BP fall [26]. Of note, all long-acting antihypertensive drugs, independently of their mechanisms of action, as well as renal denervation lower BP during night-time and reduce the early morning BP rise.

From a pathophysiological point of view, this mechanism can be explained as follows: in situations of low glomerular filtration rate, renal hypoperfusion, or increased tubular sodium reabsorption due to a variety of reasons, as well as in case of inappropriately increased dietary sodium, individuals may experience daytime sodium retention, leading to the need of an increase in night-time BP to excrete sodium (through pressure-natriuresis mechanism) and thereby maintain balance. Interestingly these mechanisms play possibly a role in favouring obstructive sleep apnoea (OSA). Indeed, night-time fluid retention which is enhanced in patients with OSA and its nocturnal rostral redistribution from the legs when shifting to supine posture, may narrow the upper airway and increase its collapsibility, and thus predispose to or aggravate OSA during sleep.

Nevertheless, further research is needed to better understand the intricate relationships between sodium excretion, renal function, and circadian rhythms, particularly in the context of cardiovascular and renal diseases.

### The renin-angiotensin system

The renin–angiotensin system (RAS) is well known to be involved in sodium handling and in modulating neural autonomic cardiovascular regulation [27,28], two key factors influencing nocturnal hypertension. Even though RAS overactivity has been proposed as a potential mechanism contributing to nocturnal hypertension, evidence showing the expression of this system in this hypertensive phenotype is scarce. The angiotensin (Ang) converting enzyme D allele has been shown to be associated with increased nocturnal BP [29]. However, low levels of Ang II were reported in preeclamptic women with nocturnal hypertension, even though they were characterized by elevated BP levels [30,31]. Along the same line, individuals with isolated nocturnal hypertension displayed low circulating Ang II levels [32] as well as lower Ang-(1-7) levels than normotensive individuals [32]. Ang II and Ang-(1–7) levels were inversely correlated with night-time diastolic BP. Thus, alterations in circulating angiotensins levels appear to contribute to isolated nocturnal hypertension development. Other components of the RAS may also be involved, like Ang A, which is generated by Ang II decarboxylation and elicits pressor responses [33,34] or Ang III, produced from Ang II by aminopeptidase A and characterized by a central neural pressor action mediated by the autonomic nervous system [28]. However, evidence on a specific contribution of Ang A and Ang III to nocturnal hypertension is lacking.

Even though Ang II levels were lower in individuals with isolated nocturnal hypertension than in normotensive individuals, ACE2 activity was not different between these groups [32] Not only circulating RAS may be involved in nocturnal hypertension but also tissue RAS. Urinary angiotensinogen, that reflects intrarenal RAS activity, has been shown to be higher in patients with CKD with nocturnal hypertension [35] This underscores the role of renal dysfunction in nocturnal hypertension and its association with intrarenal RAS activation during night-time. In addition, the activation of the intrarenal RAS during night-time, associated with nocturnal hypertension, correlates with renal arteriosclerosis in normotensive immunoglobulin A (IgA) nephropathy patients [36].

### The autonomic nervous system

Understanding the physiology and pathophysiology of nocturnal BP regulation by the autonomic nervous system is essential to address the causes of nocturnal hypertension and nondipping profile and to define specific interventions aimed at reducing cardiovascular risk associated with these phenomena. Elevated sympathetic drive emerges as a particularly relevant mechanism in this regard.

Approaches for the assessment of neural autonomic mechanisms, involved in determining the level of nocturnal BP, rely mainly on methods such as catecholamine measurement, heart rate variability, and baroreflex function tests. The last approach can rely on laboratory methods, such as the neck chamber technique to manipulate carotid transmural pressure, or the injection of vasoactive drugs such as phenylephrine and nitroglycerine to assess the reflex changes in R–R interval following drug induced BP fluctuations. It can also rely on “spontaneous” methods such as the sequence method or spectral analysis of BP and RR interval spontaneous fluctuations, which allow the assessment the spontaneous modulation of arterial baroreflex sensitivity [37]. Among the methods to quantify sympathetic cardiovascular modulation, micro-neurography stands out as the sole method directly evaluating efferent sympathetic drive to the muscles district. A major limitation of most laboratory tools lies in the fact that they primarily address sympathetic activity during wakefulness and are not easily applicable during sleep, at variance from “spontaneous” methods. Nonetheless, convincing evidence now links autonomic function to sleep-related changes in BP. For instance, a pivotal study using microneurography revealed the strict regulation of sympathetic drive during different sleep stages [38]. Laboratory studies with microneurography also identified increased levels of daytime sympathetic activity in nondippers and, especially in patients with an increase in nocturnal BP (risers) [40].

In order to investigate directly autonomic function in ambulant individuals during both wake and sleep, methods without need of laboratory manoeuvres are needed. Among them, the above-mentioned techniques based on computer analysis of BP and heart rate variability may provide deeper insights into spontaneous autonomic modulation of the heart and the peripheral vessels not only during daytime but also during night-time. Indeed, BRS evaluation based on Sequence technique analysis provided significant information on autonomic modulation during sleep both in healthy subjects and patients with sleep disorders [39,41] These methods have allowed to identify a number of clinical conditions, such as diabetes mellitus, ageing and arterial hypertension, characterized by a loss of the physiological day-night modulation of BRS and of the accompanying sympathetic and parasympathetic cardiac efferent modulatory activity [42,43,44], although the accuracy of heart rate variability in estimating cardiac sympathetic activity remains matter of debate.

On the other hand, progress in more direct methods of sympathetic activity assessment such as the measurement of skin sympathetic nerve activity allowed to demonstrate its role in determining nocturnal BP fall in an ambulatory setting in postural tachycardia syndrome patients [45].

## CURRENT METHODS OF NOCTURNAL BP ASSESSMENT

Initially, the research on nocturnal BP relied on continuous BP monitoring techniques, either invasive or noninvasive (e.g., Portapres device) [8,46]. However, these methods, while having the advantage of identifying rapid BP fluctuations, are not easily applicable outside laboratory settings. Because of that, the assessment of nocturnal BP in clinical practice currently relies on the information provided by intermittent 24-h brachial cuff ABPM. This technique, however, has important practical limitations. Therefore, new approaches to BP monitoring in this field are being developed and investigated. Whatever the BP monitoring technique employed, however, the accuracy of nocturnal BP assessment importantly depends on the method selected to identify the wake and sleep time periods.

### Defining nocturnal BP

**FIGURE 1:**
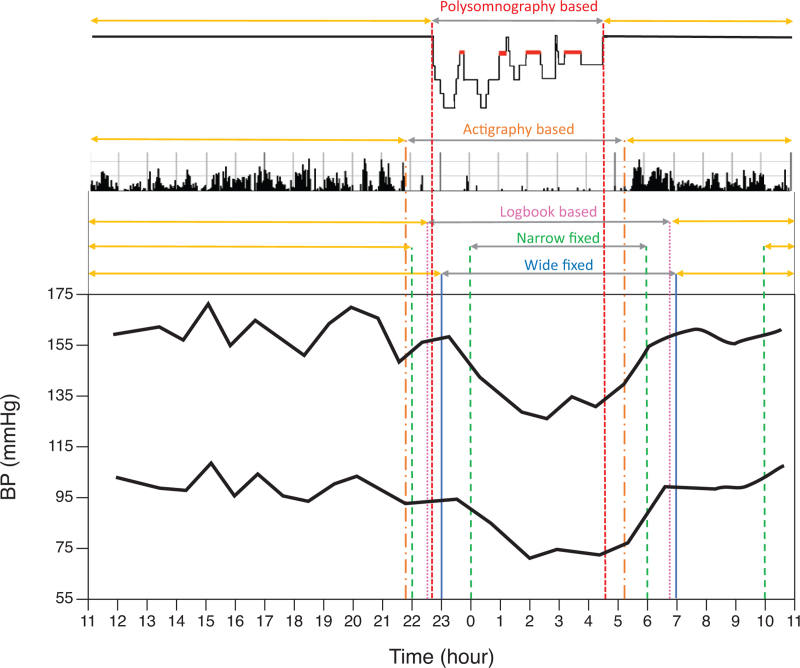
Schematic representation of different approaches to identify sleep/bedtime and awake/active time.

Several methods have been proposed over the years to identify the nocturnal period, with different accuracy, including: “wide-fixed” intervals, corresponding to the “average” expected activity and sleep patterns in a given population (e.g., Night-time 23–7 h and daytime 7–23 h in most Western countries); “narrow-fixed intervals (i.e., intervals defined by removing the transition periods between wake and sleep, e.g., day 10–22 h, night 0–6 h); definition of daytime and night-time based on self-reported information provided by the patient with the activity diary or log-book entries; actigraphy to identify body movement and position; full sleep monitoring by polysomnography, including electroencephalography to precisely identify sleep occurrence (Fig. 1).

These approaches may give a good approximation of “nocturnal” period in most patients but it has been shown that its definition can vary significantly when based on either: night-time (clock time corresponding to the night); time in bed (time when an individual is resting in bed); and sleep time (time when an individual is actually sleeping).

While these different approaches may significantly affect estimates of nocturnal BP and dipping in individual subjects [47,48], their possible different relevance in terms of outcome prediction has not been demonstrated [49].

**FIGURE 2:**
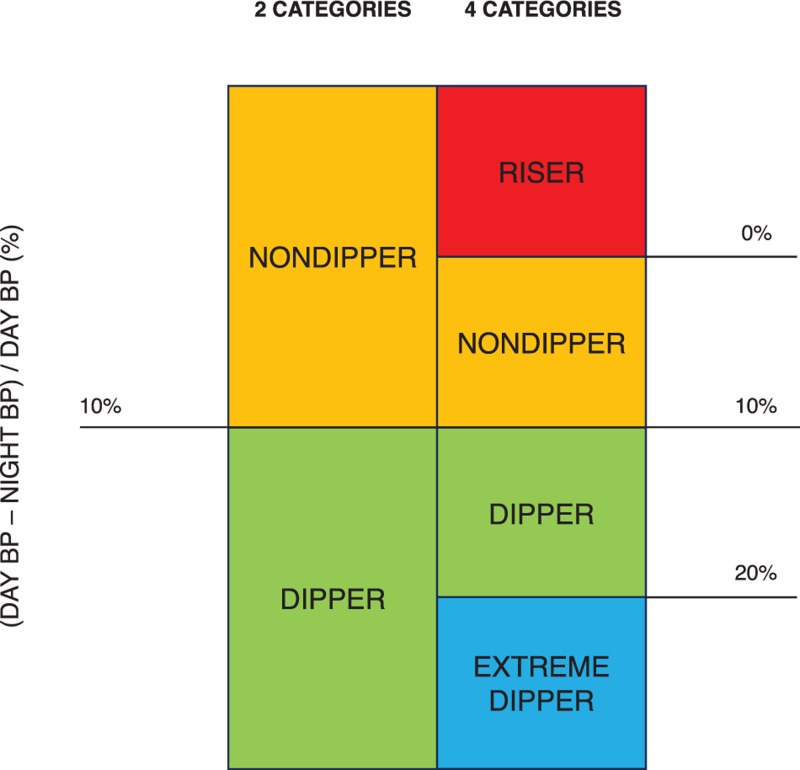
Dipping classification based on two or four categories.

Different approaches are also used to describe the behaviour of nocturnal BP. It can be viewed as a continuous variable or categorized into normal and elevated, with nocturnal hypertension being defined in the 2023 ESH Hypertension Guidelines as average nocturnal SBP ≥120 mmHg and/or nocturnal DBP ≥70 mmHg). It can be also assessed in relation to daytime BP by calculating the extent of nocturnal BP fall (dipping). According to the presence and to the size of BP dipping, arbitrary categories such as dippers, nondippers, extreme dippers and risers can be defined (Figure 2).

### Discontinuous 24-h ambulatory blood pressure monitoring

For the moment, 24-h ABPM performed with validated automatic upper-arm cuff devices is the cornerstone of nocturnal BP measurement. The general principles of ABPM methodology have been delineated elsewhere [50]. The conventional cuff-based approach poses, however, difficulties for BP monitoring at night, including tolerability issues, artefacts, reproducibility and accuracy concerns [51,52].

Many individuals poorly tolerate conventional 24-h ABPM, mainly due to discomfort caused by repeated arm cuff inflations. This aspect may be of particular relevance during the night because BP measurement may induce arousals with difficult-to-predict effects on BP of the individual [53,54].

When focusing on day-night BP changes, also issues mostly affecting daytime measurements should be considered, such as the artefacts due to motor activity. Additionally, artefacts from arrhythmic events during measurement may impact algorithm interpretation. In one study over 26% of measurements were affected by such artefacts [51]. Daytime motor activity predominantly caused artefacts, while nocturnal arousals related to cuff inflations were significant during sleep, affecting night-time BP levels. Correcting for artefacts led to a substantial reduction in BP variability estimates and to changes in hypertension classification in a considerable number of participants [51]. This contributes to explain the observation that, while 24 h average ABP values have excellent reproducibility at the population level, the reproducibility of ABP values averaged over subperiods of the 24 h, and particularly the reproducibility of the dipping status at the individual level is limited [52].

Finally, in conditions such as OSA or restless legs syndrome, discontinuous BP monitoring may not be able to capture rapidly occurring BP changes over seconds or minutes due to these sleep-related disorders.

Despite these challenges, cuff-based ABPM has demonstrated overall good clinical usability and good ability to predict outcomes. Thus, while acknowledging its methodology-related limitations, ABPM remains a vital tool for clinical practice recommended in all major hypertension guidelines, and is still the preferred option for nocturnal BP monitoring, supported by evidence on its prognostic value.

Some of the currently available ABPM devices are equipped with algorithms for estimating central BP. A recent report in a large cohort of untreated adults indicated that nocturnal fall may be less pronounced for the estimated central BP than for peripheral BP, especially in young individuals. The accuracy of central BP estimated by these algorithms and the clinical meaning of these findings remain to be further explored [55].

#### Home or ambulatory BP monitoring?

Although ABPM remains the reference method to evaluate nocturnal BP, novel self-measurement home BP devices equipped with nocturnal BP measurement function have been developed, with the first nocturnal home BP monitor device introduced in 2001 [56]. A recent meta-analysis of studies comparing nocturnal home BP monitoring (HBPM) vs. classic ABPM [57–62] showed that nocturnal home BP monitoring, when compared to nocturnal BP monitoring by 24 h ABPM. presents similar values, has reasonable agreement in detecting nocturnal hypertension and nondipping, as well as comparable relationship with indices of preclinical organ damage [63]. Furthermore, recent prospective outcome data showed that uncontrolled nocturnal hypertension defined by HBPM (independent of office SBP) predicts future cardiovascular events similarly to classic nocturnal hypertension detection by ABPM [64].

Similarly, a very recent paper by Tabara *et al.* showed that in 5814 community residents of the Nagahama study followed up for 7.3-years nocturnal HBP was independently associated with the incidence of cardiovascular disease [65]. Compared to other studies on nocturnal BP, this study has the merit that nocturnal HBP reflected presumably sleep BP as participants were asked to wear actigraphy during the study. Furthermore, the authors could at least in part exclude the confounding factor of sleep disordered breathing as pulse oximetry for the first four nights was also evaluated. In the real-life application however we need to take into account that several confounders may reduce the accuracy of HBPM results, in particular comorbid insomnia (in particular COMISA = obstructive sleep apnoea + insomnia), restless legs syndrome but also positional OSA or REM-related OSA which can be underestimated by pulse oximetry

A relevant aspect to be considered when using HBPM is the nocturnal BP monitoring schedule. Preliminary evidence suggests that a total of six readings obtained in two successive nights (3 readings/night) seems to be the minimum requirement for clinically useful information [66,67]. Patient preference is also of paramount importance when considering the choice of ABPM or HBPM, and data suggests that nocturnal HBPM is preferred to classic ABPM due to less sleep disturbance [58].

Thus, HBPM for nocturnal BP evaluation is feasible, and is reported by a few studies to provide similar values as 24 h ABPM. However, further research is needed not only to further refine the methodology of its clinical use, but also to explore the potential applications, benefits, and limitations of these HBP devices. Available evidence seems to support their practicality and cost-effectiveness as patient-owned devices, suggesting that they are probably more appropriate for repeated nocturnal BP evaluation also in the long term.

### Wearables and multiparametric sleep monitoring

Wearable devices designed for the consumer market, able to measure and monitor several health-related variables are now widespread, owing to the stunning technological progress which led to considerable miniaturization of sensors and reduction of costs. Some of these devices provide BP estimates, also during sleep. This expanding field is driven by the medical technology and nonmedical technology industry. When addressing their application to nocturnal BP monitoring, the key question in this regard is: what would the desirable attributes for wearable technologies aimed at monitoring nocturnal/asleep BP be? One crucial attribute is noninvasiveness and no disturbance of sleep quality, ensuring that the monitoring process does not interfere with the physiological parameter it seeks to measure (e.g. causing arousals). Indeed, minimal intrusion is vital to avoid disrupting the measurement procedure. Ideally, these technologies should provide frequent measurements, acknowledging the continuous nature of BP rather than relying on intermittent readings. From this point of view, cuffless BP monitoring devices are particularly promising, as they eliminate cuff-related intrusion and disturbance to physiological parameters, and may even allow for continuous monitoring in some cases.

On top of all requirements, however, these monitors must ensure acceptable accuracy. Cuff-based measurements rely on surrogate signals like sounds or oscillations closely related to the pressure in the underlying artery. Conversely, most cuffless devices rely on mathematical models integrating several physiological parameters, rather than measuring the forces more directly related to BP. To ensure acceptable accuracy, cuffless devices should demonstrate comparable accuracy to gold-standard, cuff-based devices and be validated in accordance to internationally recognized protocols and recommendations, focusing not only on their accuracy in quantify BP levels, but also on their ability to reliably track BP changes over time [68].

Thus, while various techniques are available, not all of them can reliably derive nocturnal BP: Table 2 summarizes the different methods adopted and their advantages and disadvantages in relation to nocturnal (asleep) BP measurement.

**TABLE 2:** Summary of different methods for nocturnal BP measurement

Category	Method	Nocturnal BP measurement availability	Advantages for nocturnal BP measurement	Disadvantages for nocturnal BP measurement
ABPM	Oscillometric	Yes	Solid evidence that correlates nocturnal BP with outcomes	can disturb sleep, suboptimal reproducibility, not widely available, relatively expensive; difficult to obtain information on more than one night
Home BP	Oscillometric	Yes	May disturb sleep less than ABPM and allow multiple measurements over several different days	Little evidence that correlates nocturnal HBP with outcomes, suboptimal concordance with ABPM-derived nocturnal BP
Calibrated cuffless wearables	PTT (PAT)	Yes	Solid theoretical basis. Regulatory approval of brachial cuff-calibrated devices	Need periodic cuff-based calibration. Compelling published data are still limited. Practical PTT measurements have yet to convincingly show high intraindividual correlations with reference BP measurements.
	PWA (PPG)	yes	Potentially based on a single sensor. Regulatory-approved cuff-calibrated devices	Less robust theoretical underpinnings. Compelling published data are still limited. Need periodic cuff-based calibration
Uncalibrated cuffless wearables	Volume control	yes	continuous BP measurement	Some of them can create discomfort at night due to finger numbness few published studies

Adapted from [69]. BP, blood pressure; PP, pulse pressure; PPG, photoplethysmography; PTT, pulse transit time; PAT, pulse arrival time; PWA, pulse wave analysis.

This field is in constant evolution. However, solid and unbiased clinical evidence on the accuracy of cuffless devices is still lacking making it difficult to adopt them in clinical practice. A Microsoft research project attempted to compare wearable devices showing the potential advantages of waveform feature models, even without direct measurements [70]. Of note, this same project highlighted the still limited value of cuffless methods based on pulse arrival time [71]. Overall, while these devices show promise and reflect the evolving landscape of medical technology, empowering patients with accessible tools for monitoring, their accuracy still presents considerable problems, as highlighted by an ad hoc position paper of the ESH Working Group on Blood Pressure Monitoring and Cardiovascular Variability [72] In particular, the ability of cuffless devices to accurately track BP changes in time was questioned. The most evident problem in this regard was the underestimation of nocturnal BP fall, a pitfall that appears crucial in the field of nocturnal BP assessment [73], [74] although improvements in this regard were reported [75,75].

These concerns emphasize the need for independent clinical validation studies based on internationally accepted protocols. A proposal for such a protocol has been published by a group of international experts to account for the different designs and applications of cuffless devices [68]. Future research will have to show whether novel approaches to BP measurement will be able to satisfy the required criteria and therefore demonstrate their safety and clinical usability.

Additionally, the clinical applicability of the data provided by these devices is unclear and depends on how the specific model is designed: some of them only provide information similar to a conventional discontinuous ABPM, while others may report BP-related data with high frequency (or even continuously), combined with other parameters (ECG, blood oxygenation, body movements etc.). In the latter case their interpretation may require elevated expertise, especially when they are used to evaluate BP modulation in patients with sleep disorders. For this kind of use it is mandatory to collect a detailed sleep history, whenever possible accompanied by a quantitative sleep assessment, especially if the information is used to optimize cardiovascular risk assessment and treatment decisions.

In conclusion, the quest for accurate nocturnal (asleep) BP monitoring through wearables is a formidable challenge. Multimodal approaches, including parameters related to cardiac effects and heart rate variability, may show promise in improving the accuracy of measurements. As the field evolves, balancing accuracy, precision, and individual variability will be crucial for the successful implementation of these technologies in healthcare.

## CLINICAL IMPLICATIONS

### Implications for hypertension diagnosis (masked hypertension due to isolated nocturnal hypertension)

Historically, hypertension has been diagnosed for many years only based on office BP measurements, but with the introduction of out-of-office measurements like ABPM or HBPM, discrepancies with office BP in terms of patients’ classification emerged. This is when white-coat hypertension and masked hypertension were recognized as distinct BP phenotypes in untreated patients. Subsequently, the terms masked uncontrolled hypertension (MUCH) and white-coat uncontrolled hypertension (WUCH) were proposed as more accurate for treated patients, considering treatment effects and implications for clinical management.

Recent international guidelines acknowledge the role of out-of-office BP measurements and define white-coat and masked hypertension based on discrepancies between office and out-of-office BP measurements [76,77]. However, while US guidelines define masked hypertension focusing on daytime ABPM or HBPM for untreated patients and propose only vague criteria for treated patients [76], the European guidelines also mention MUCH in treated patients and indicate the usefulness of 24 h ABPM, including measurements during sleep. Neither US nor European guidelines clarify whether elevated out-of-office BP should be assessed by HBPM or ABPM [77]. This is relevant in the context of nocturnal hypertension, which in current clinical practice can only be identified through ABPM. The two techniques are not interchangeable for identifying masked BP: although they provide similar risk estimates [78], their diagnostic agreement is low.

**FIGURE 3:**
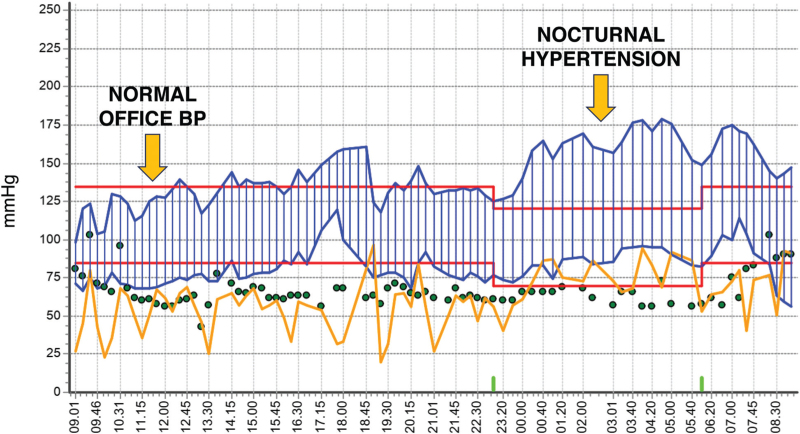
Example of 24 h ambulatory blood pressure recording displaying a case of masked isolated nocturnal hypertension in a patient affected by obstructive sleep apnoea (OSA).

One of the reasons for the above finding might be the presence of different masked BP phenotypes with different underlying mechanisms: daytime masked hypertension may involve sympathetic nervous system activation, particularly in stressful working environments [79,80], while nocturnal masked hypertension (frequently equivalent to isolated nocturnal hypertension) may be due to specific conditions discussed in more detail in other sections of this document. Prevalence of nocturnal masked hypertension was indeed shown to be influenced by sleep-related factors [81] (see Fig. 3, showing isolated nocturnal hypertension in a patient affected by OSA), seasonal changes [82] and high-altitude residence (with excessive erythrocytosis as an independent predictor) [83].

In summary, masked hypertension presents various subtypes, which are poorly intercorrelated and may have different predictors and different impact on outcomes. Among these phenotypes, nocturnal masked hypertension is not uncommon and is especially challenging to diagnose but holds both diagnostic and prognostic significance, necessitating further research.

### Relationship with organ damage

Nocturnal BP patterns are associated with target organ damage in patients with hypertension: Studies on patients with essential hypertension have indeed shown a correlation between elevated night-time BP or a nondipper BP profile and a higher risk of damage to target organs, including the heart, kidneys, blood vessels, and brain [84–86].

As mentioned above, while the assessment of the 24 h BP profile by ABPM is not feasible in all patients, new devices for the assessment of home BP might allow to estimate nocturnal hypertension more easily. Within the J-HOP study, nocturnal hypertension was prevalent, and its association with target organ damage was consistent regardless the device used (ABPM or HBPM) [59]. However, while masked nocturnal hypertension was associated with increased risk of cardiovascular events (see next paragraph) and demonstrated varying prevalence among seasons, similar risk profiles were observed for organ damage [87].

Considering that current methods of measuring night-time BP are not readily available in all patients, a predictor score for identifying those in whom nocturnal BP may be elevated was proposed in order to identify candidates for nocturnal BP monitoring. The predictors included male sex, high BMI, diabetes, elevated office and home BP and elevated urinary albumin-creatinine ratio [90]. However, the generalizability of this score beyond the Japanese population requires further validation.

Examining the impact of bedtime dosing of antihypertensive drugs on BP reduction at night revealed no significant difference as compared to morning dosing, but controlling night-time BP was associated with reduced target organ damage in the J-TOP study [91].

In conclusion, night-time BP exhibits associations with target organ damage independently of daytime measurements. This underscores its clinical significance and the potential need for specific populations to undergo aggressive evaluation and management of night-time BP, an indication which requires to be addressed by further studies.

### Relationship with cardiovascular outcomes

In 1988, O’Brien *et al.* introduced the term ‘nondippers’ and demonstrated a significantly higher frequency of stroke in individuals with nocturnal BP increase [92]. Subsequent studies have confirmed that elevated night-time BP is a strong predictor of cardiovascular events. In the Ohasama study individuals with nondipping and elevated night-time BP had a significantly higher risk of cardiovascular mortality [93].

Data from the IDACO project revealed that in treated hypertensive patients undergoing 24 h ABPM, only night-time BP levels were independently associated with cardiovascular events, while daytime levels were not [94]. This underscores the importance of measuring night-time BP in patients with hypertension, as it may be crucial for their prognostic assessment. This is also the case for nocturnal home measurements: in J-HOP study both nocturnal masked hypertension and nocturnal sustained hypertension identified with this approach were associated with worse outcome compared with individuals with normal nocturnal BP [95].

Data from the JAMP study showed an independent association of night-time BP levels and a riser pattern with cardiovascular events, particularly heart failure [88]. The data from the J-HOP study support this association demonstrating that BNP levels, indicating increased circulating volume, are increased in patients with increased night-time BP, supporting the hypothesis that night-time BP is associated with blood volume dysregulation [89].

However, differences in outcomes might be related to different BP profiles: data from the Ohasama study have shown that night-time BP levels are significantly associated with total cerebral and cardiovascular mortality. Notably, for ischemic stroke, only night-time BP was a significant predictor, while for haemorrhagic stroke, daytime BP was more predictive [96].

The number of measurements to evaluate nocturnal BP might also play a role in the association with CV outcomes. International guidelines recommend reporting daytime and night-time BP measurements and the number of the latter should be ≥7 according to ESH guidelines. Nevertheless, data from the IDACO study indicate that increasing the number of measurements to 8 at night can enhance predictive value [97]. While a minimum of four night-time BP readings might be acceptable, more readings provide a preferable and more accurate assessment.

Sex differences should be also mentioned: data from the IDACO database revealed that men generally had a higher absolute risk of total mortality and cardiovascular events in relation to elevated ABP levels. However, the relative risk reduction associated with a decrease in 24-h or night-time BP was more pronounced in women, especially for night-time readings [98]. This suggests a significant potential for cardiovascular prevention through BP-lowering treatment in women.

In summary, the impact of night-time BP on cardiovascular outcomes is pronounced in treated hypertensive individuals, varies between ischemic and haemorrhagic strokes, benefits from a higher number of measurements, and shows gender-specific differences in relative risk reduction (Box 2).

Box 2 **Clinical implications of nocturnal hypertension and nondipping pattern**

**Table:** 

Relationship with Organ Damage • Elevated nocturnal BP and nondipper BP patterns are linked to increased target organ damage, affecting the heart, kidneys, blood vessels, and brain. • The J-HOP study found that nocturnal hypertension measured by home or ambulatory BP monitoring (HBPM or ABPM) consistently correlated with target organ damage. Relationship with Cardiovascular Outcomes • Elevated night-time BP is a strong predictor of cardiovascular events, with the Ohasama study showing a higher risk of mortality in individuals with elevated nocturnal BP. • The IDACO project highlighted that only night-time BP levels in treated hypertensive patients were independently associated with cardiovascular outcomes, emphasizing the prognostic importance of nocturnal BP monitoring.

### Nocturnal hypertension vs. nondipping

More than half a century has passed since the first demonstration that in normal individuals arterial BP experiences a roughly 10–20% drop from day to night [15]. Since then, numerous studies have been published investigating the association between daytime BP and the BP fall from day to night. A reduction of the size of the day-night BP difference, may in principle be driven either by elevated nocturnal BP level or by lower daytime BP level. The role of nocturnal BP level appears prevalent in this regard, although variations in daytime BP, for example caused by different degrees of physical activity, may contribute as well [99].

**FIGURE 4:**
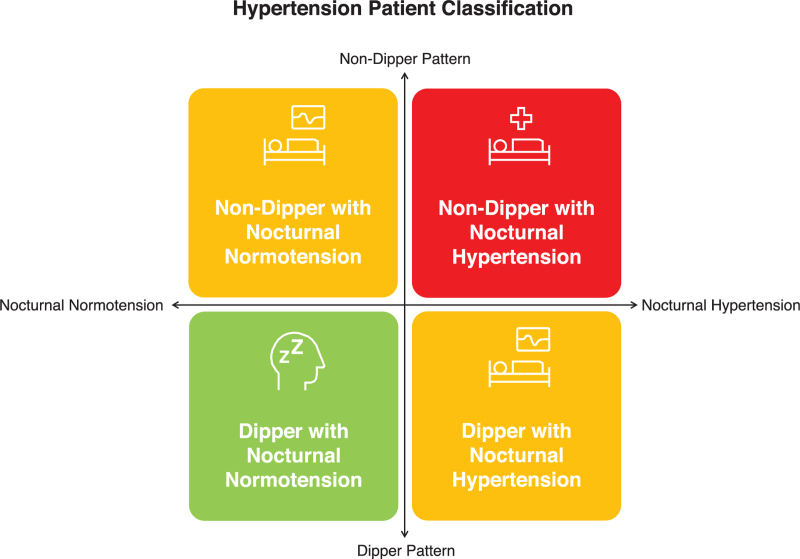
Different possible associations between dipping and nocturnal hypertension.

It has to be remembered that non dipping and nocturnal hypertension, while frequently coexisting, are two different phenomena. The former indicates altered circadian BP modulation and the latter absolute elevation of BP at night (regardless of daytime values; see Fig. 4). The question of whether clinical decisions should be preferably based on average nocturnal BP/nocturnal hypertension or on nocturnal dipping has not been clearly settled and may depend on the clinical problems underlying these conditions. Abnormal findings in this regard may lead to active search for underlying pathologies (e.g., sleep apnoea, endocrine disorders etc.), may be used to refine the estimation of BP-related risk and, finally, may encourage the physician to modify antihypertensive treatment.

Whatever the application, assessment of the reproducibility of these phenotypes is highly relevant. In a recent meta-analysis on short-term ABPM reproducibility although the group average size of nocturnal BP fall did not change between two 24 h recordings, 32% of participants changed dipping category [100]. Even within a 48-h ABPM this percentage remained elevated with 24% of participants switching category between the two nights [101]. This problem was even more relevant if four-category dipping classification was used [102] Conversely, the reproducibility of nocturnal BP level is better and comparable with that of daytime BP when assessed by ABPM [103] or by home BP [104] When patients are categorized by nocturnal hypertension/normotension status, 18% were reported to change category on repeated ABPM [105]. The source of limited reproducibility of day-night BP change may depend on the variable activity patterns but also by sleep disturbance induced by the measurement procedure itself. In fact, the dippers/nondippers classification becomes less associated with outcome in hypertensive patients who report feeling sleep-deprived for >2 h during the course of the overnight monitoring [54].

When considered separately both night-time BP levels and day-night BP dip are significantly associated with cardiovascular outcomes. This holds true across various settings, including the general population [106] and hypertensive patients [107]. Data from different studies indicate the superior prognostic value of night- time BP compared not only to office BP but possibly also to daytime ambulatory BP, especially in treated individuals [94,108,109]. In the Spanish ABPM registry reduced nocturnal BP fall was associated with cardiovascular mortality even after adjusting for 24-h BP and remained predictive also among the subgroup with normal nocturnal BP (“normotensive nondippers”) with a HR of 1.17[95%CI1.00–1.35]. The presence of nocturnal hypertension with preserved dipping was characterized by an even stronger association (hazard ratio (HR) 1.62 [1.39–1.90]) and effect on the prognosis was most pronounced when nondipping and nocturnal hypertension coexisted (HR 1.90 [1.68–2.14]) [110].

In conclusion, both night-time BP and the day-night BP dip are robust and partly independent predictors of cardiovascular risk although nocturnal BP level appears to be superior in this regard. The utility of BP dip classification remains uncertain, also due to its low reproducibility, especially in patients reporting significantly disturbed sleep during monitoring. Therefore, the clinical interpretation of nondipping should take into account sleep quality and repeated ABPM should be considered to confirm abnormal nocturnal BP patterns.

## SPECIAL POPULATIONS

### Neurological disorders

Neurogenic supine hypertension represents a key sign of autonomic failure that is often due to autonomic neuropathy. The latter can be secondary to chronic conditions such as diabetes and paraneoplastic syndromes or is defined as “primary” when due to inherited or neurodegenerative diseases affecting the autonomic nervous system, including synucleinopathies such as pure autonomic failure, multiple system atrophy, and Parkinson's disease [111,112].

The prevalence of supine nocturnal hypertension is around 50% in primary autonomic neuropathy [112], and its pathogenesis involves factors such as baroreflex failure and increased vascular resistance [113]. It is often associated with nocturnal hypertension and the diagnosis involves measuring BP in the supine position and performing 24 h ambulatory BP monitoring. The clinical features include silent hypertension, nocturia, and low orthostatic tolerance. Supine hypertension is associated with long-term consequences such as increased risk of heart failure and cardiac-cerebrovascular death [114] and target organ damage [115].

Treatment is challenging, with a priority on managing orthostatic hypotension due to short-term risks. Nonpharmacological approaches include head-up tilt nocturnal rest, evening snacks to induce postprandial hypotension, and avoiding antihypotensive drugs, ingestion of abundant water and elastic stockings in the evening. Local heat and CPAP have been also shown to be effective but with a low level of evidence [116,117]. Pharmacological therapy requires individualization, considering the risk-benefit ratio. Short-acting antihypertensive drugs at bedtime (including nitroglycerine patch, short acting ACEI/ARBs, eplerenone, sildenafil, or clonidine) may be proposed [118,118], whereas long-acting drugs like alpha-blockers and diuretics should be avoided. Careful drug selection (beta-blockers can reduce the minimal chronotropic response to orthostatism), and the use of ABPM for timing are suggested.

In conclusion, supine nocturnal hypertension in autonomic neuropathy is linked to organ damage, and its diagnosis is straightforward. Treatment decisions should weigh short-term risks of orthostatic hypotension against the long-term risks of supine nocturnal hypertension, and a personalized approach is crucial due to the lack of clear guidelines and approved medications for this specific condition.

### Patients with sleep disorders

Interest in sleep disorders in relation to cardiovascular disease has been stimulated by the evidence that night-time BP is a more informative predictor of mortality than clinic BP. Furthermore, dysfunction in 24-h circadian rhythm is a common occurrence in ageing adults, more severe in people with age-related neurodegenerative diseases, including Alzheimer's disease and related dementias, and Parkinson's disease, which are also associated with sleep problems [119]. Chronic sleep deprivation and sleep disorders, such as insomnia, restless legs syndrome-periodic leg movements, and obstructive sleep apnoea, are highly prevalent, potentially contributing to the abnormal BP pattern at night and ultimately to increasing incidence of hypertension and early mortality [120,121].

Sleep apnoea is characterized by repeated episodes of partial or complete pharyngeal collapses during the night, often due to the narrowing of the upper airway.

Fluctuations in BP during such episodes, first reported by Coccagna *et al.*[122] and later confirmed by Guilleminault at Stanford University [123], are primarily attributed to intermittent hypoxia and sympathetic activation. These episodes lead to peripheral chemoreceptor stimulation, resulting in enhanced sympathetic activation, production of reactive oxygen species, systemic inflammation, and impaired endothelial function. Additionally, arousals from sleep and increased pleural pressure swings further stimulate the autonomic nervous system. Studies have shown that patients with OSA exhibit increased catecholamine levels and sympathetic neural traffic. Further supporting these mechanisms, in treated OSA patients, the withdrawal of OSA treatment has been shown to be associated with an increase of the activation of the sympathetic nervous system [124].

Epidemiological studies indicate a significant occurrence of sleep apnoea, especially in older age groups, with a potential link to hypertension [125,126]. Research suggests that even a low number of apnoea events can increase the risk of hypertension, with a stronger association in younger individuals [127,128]. Accordingly [129], hypertensive patients, particularly those with resistant hypertension, often exhibit a high prevalence of sleep apnoea [130,131]. The mechanisms behind this association, as previously mentioned, involve factors like intermittent hypoxia, sympathetic nervous system activation, and endothelial dysfunction [132,133,134].

The neurological mechanisms linking sleep disorders, lifestyle habits, and sympathetic activation play a key role in favouring inflammation, insulin resistance, oxidative stress, and hypertension [135]. Various studies, including a systematic review, highlight the association between sleep disorders and increased night-time BP, both systolic and diastolic [136,121]. Additionally, as shown in a sub analysis of the Sleep Heart Health Study, the combination of different sleep disorders, like insomnia and sleep apnoea, results in higher cardiovascular and metabolic risks, emphasizing the need for comprehensive treatment approaches [137].

In addition there is growing data on 24 h blood pressure control impairment in patients with REM behaviour disorder which is acknowledged as an early prodromal stage of synucleinopathy since it has been described in individuals with a high risk of developing neurodegenerative diseases, particularly synucleinopathies [138].

In conclusion, recognizing poor sleep quality and overall sleep disorders as contributors to hypertension, especially nocturnal hypertension, is crucial. Physicians should consider sleep as a modifiable risk factor for hypertension, advocating for comprehensive treatment strategies that address both sleep disorders and traditional interventions for hypertension. Notably, recent recognition of sleep as a significant risk factor for cardiovascular health underlines the importance of incorporating attention to sleep problems into overall health management [139] (Table 3).

**TABLE 3:** Potential mechanisms linking sleep disorders with abnormal diurnal BP profile

Sleep disorder	Pathophysiological mechanism
Obstructive sleep apnoea	Repetitive episodes of intermittent hypoxia and arousals from sleep due to partial or complete upper airway occlusion increase sympathetic activity during sleep, determining nocturnal hypertension and nondipping pattern in association with endothelial dysfunction, systemic inflammation and changes in intrathoracic pressure.
Insomnia	insomnia and its related night-time autonomic hyperarousal, may entail blunted sleep-related BP dipping due to factors including arousals and reductions in sleep time, which would lead to phasic or tonic increases in sympathetic vasoconstrictor activity, respectively
Restless legs syndrome	Reduction in sleep time may contribute to alter night-time BP control also in subjects with RLS. Furthermore, Periodic limb movements during sleep (PLMS, which are common in RLS) and their related arousals, might lead to phasic increases in BP
Central hypersomnias	Abnormal diastolic night-to-day cardiovascular regulation is present in patients with narcolepsy and cataplexy and is mainly associated with sympathetic activation due to instability of REM sleep and to the circadian sleep wake rhythm derangement
REM behaviour disorder	Altered diurnal BP profile and neurogenic orthostatic hypotension appear to be related to autonomic dysfunction, common in synucleinopathies associated with RBD. In addition, an anatomical link is likely, given the close proximity between the REM sleep and autonomic control nuclei in the brainstem.

### Relationship with kidney disorders

The correlation between nocturnal BP, hypertension, and CKD has long been recognized. The assessment of night-time BP is particularly crucial for various patient groups, including those with CKD as highlighted in a EURECA-m working group position paper [140,141]. The discussion on CKD and its relationship with night-time BP has prompted changes in relevant clinical guidelines that currently recommend the use of ambulatory or home BP monitoring to complement office measurements in CKD patients, emphasizing its importance in the diagnosis and management of hypertension, in particular in dialysis patients [142].

The mechanisms of altered nocturnal BP profile in CKD include the increase in sympathetic drive during night-time, a reduced renal sodium excretory ability, sleep-disordered breathing such as OSA, leptin and insulin resistance, endothelial dysfunction and glucocorticoid or cyclosporine use (in transplanted patients).

Data from the Vigo study [143], further confirmed in the Spanish ABPM registry [144] indicate distinct night-time BP profiles in CKD patients. In particular, with advancing stages of CKD the proportion of individuals with normal dipping profile progressively decreases, while the proportion of reverse-dippers progressively increases, further highlighting the need of 24 h ABPM in CKD patients. Dialysis patients, exhibit peculiar trajectories in night-time BP; with haemodialysis patients in particular showing significant BP elevations from the first to the second night of the standard interdialytic interval [145]. Another important group of patients that deserve to be mentioned are kidney transplant recipients; compared to haemodialysis patients, they exhibit a lower night-time BP despite persistence of nondipping profiles [146]. However, when they are compared to CKD patients matched for eGFR, their night-time BP and dipping patterns are generally the same [147].

The increased BP variability observed in CKD patients makes the diagnosis of hypertension challenging: BP phenotypes in CKD reveal a shift from white-coat hypertension to masked hypertension, with an increased proportion of nondippers, particularly in transplant recipients [148]. Elevated night-time BP is the main reason for the very high rates of masked hypertension in these individuals [149].

The importance of studying night-time BP in CKD is underscored by its association with cardiovascular events, disease progression, microalbuminuria, and mortality [150]. Prognostic associations emphasize the utility of night-time BP in predicting outcomes, with nocturnal BP being a stronger predictor than daytime BP [150].

In conclusion, night-time BP is a critical parameter in understanding and managing hypertension in CKD patients. Its association with various outcomes highlights its prognostic value, and ongoing research, especially considering sex differences, contributes to a deeper understanding of its clinical implications.

### Relationship with metabolic and endocrine disorders

Circadian BP profile is associated with BMI through several mechanisms including enhanced sympathetic activation, the presence of sleep disorders, alterations of sodium handling, etc. Data from the Spanish Society of Hypertension Ambulatory Blood Pressure Monitoring Registry show that patients with a nondipping profile exhibited on average a higher BMI compared to those with a dipping profile, irrespective of treatment status [151]. The prevalence of obesity was significantly higher in nondipping patients, emphasizing the interplay between weight-related factors and day-night BP patterns.

Among the causes of nondipping pattern, the impaired renal capacity to excrete sodium plays an important role not only in CKD patients but also in patients with diabetes and metabolic syndrome that exhibit an enhanced tubular sodium reabsorption. In a study by Hermida *et al.*, metabolic syndrome was associated with elevated asleep systolic BP and reduced systolic BP dipping, with waist circumference identified as the most important determinant among key factors of metabolic syndrome [152]. In a recent observational study, body weight, BMI, but also lean body mass and abdominal-fat-to-total-fat-mass ratio were associated with nondipping profiles, emphasizing also the impact of body composition and fat distribution on 24-h BP pattern [153].

As mentioned previously, diabetic patients show higher asleep diastolic BP and reduced systolic BP dipping, contributing to an increased prevalence of nondipping profiles [154]. Furthermore, reverse dipping in diabetic patients was associated with autonomic neuropathy and increased mortality rates [155,156].

Other endocrine disorders have been associated with abnormal circadian BP profiles: aldosterone-producing adenoma for instance, the most prevalent form of primary aldosteronism, exhibited not only higher daytime BP but also and even more importantly an elevation of night-time BP, leading to a lower prevalence of dippers [157]. Among the mechanisms causing BP nondipping in primary aldosteronism sodium balance seems to play a key role: in a small study in patients with unilateral adenoma, BP diurnal patterns were altered in patients with primary aldosteronism who maintained a relatively high sodium intake whilst both adrenalectomy and sodium restriction restored a physiologic nocturnal dip [158].

Patients with Cushing's Syndrome also exhibit an altered circadian BP profile due to disrupted cortisol discharge, with surgical intervention gradually restoring nocturnal BP decline [159,160].

Similarly, patients with secondary hypertension due to pheochromocytoma demonstrated a nondipping pattern, associated with increased target organ damage [161]. Again, surgical treatment restored normal circadian BP pattern [162].

Understanding the intricate relationships between endocrine disorders and 24 h BP patterns is crucial for prognostication and tailored therapeutic interventions. Further research is needed to unravel the detailed mechanisms governing these complex associations and their implications for clinical outcomes.

## THERAPEUTIC ASPECTS

### Pharmacological antihypertensive treatment: role for chronotherapy?

One of the key clinical questions regarding nocturnal BP is whether bedtime administration of antihypertensive drugs can normalize night-time hypertension or nondipping patterns, ultimately improving outcome. Several meta-analyses have explored the effectiveness of morning vs. evening dosing in reducing BP levels [163,164]. While evidence suggests a significant effect on night-time BP levels in favour of bedtime administration, methodological issues in the included studies necessitate cautious interpretation. These methodological issues include: 1) arbitrary clock time or individual's self-reported awake and a sleep time; 2) lower number of night-time/asleep than daytime BP measurements; 3) different study designs; 4) lack of information on treatment allocation procedure in randomized controlled studies; 5) different sample characteristics; 6) different comparators and 7) having most data derived from a single research centre.

The HARMONY, a small crossover randomized trial including 95 patients on stable antihypertensive treatment (predominant use of long-acting formulations of agents) showed that there was no difference in terms of 24 h, daytime and night-time ambulatory BP between patients randomized on morning or evening dosing [165].

Similarly, a 26-week multicentre randomized double-blind study evaluating the efficacy and safety of valsartan 320 mg, dosed a.m. or p.m., vs. lisinopril 40 mg (a.m.) found no differences in terms of ambulatory BP levels [166].

Recent systematic meta-analyses investigated the cardiovascular prognosis of bedtime vs. morning drug administration analysing randomized trials only and showed that evening dosing of antihypertensive drugs significantly reduced ambulatory BP and cardiovascular events, although this effect was mostly driven by studies from the Spanish group of Hermida *et al.* and by studies from China, published in local journals [164,167].

Several studies assessed the impact of bedtime administration of antihypertensive treatment on outcomes. The Spanish trials MAPEC [168] and HYGIA [169] showed that evening dosing was associated with major improvement on main cardiovascular endpoints despite a modest ambulatory BP reduction. However, regarding these studies, concerns regarding methodological transparency and potential biases have been raised [170,171].

More recently three other trials were published which did not confirm these results. The TIME study included over 20,000 hypertensive patients randomized to take BP tablets either in the morning or in the evening. This pragmatic trial did not find any significant difference in cardiovascular events between evening or morning drug dosing [172]. However, since patients were randomized without knowledge of their day-night BP profile, it is unknown whether results are equally applicable in those with normal or increased nocturnal BP. Furthermore, nonadherence at any stage was high (i.e., 31%). Adherence should indeed be considered when discussing about chronotherapy: data from the TIME study confirm that taking antihypertensives in the morning was associated with better adherence (nonadherence: morning dosing 23% vs. evening dosing 39%), a factor which may outweigh possible benefits that might derive from bedtime drug intake.

Further evidence from the Chronotype sub-study of the TIME trial in over 5,000 participants suggests that the individual chronotype (early vs. late) is a strong predictor of the effect of morning vs. evening dosing of antihypertensive medications. Later chronotype is associated with an increased risk of composite end-points of nonfatal myocardial infarction, but not of stroke events [173] Alignment of dosing time with personal chronotype (evening dosing in later chronotypes and morning dosing in earlier chronotypes) could provide additional cardiovascular protection compared to a ‘misaligned’ dosing time regimen. Further studies considering the individual's chronotype may provide further insights.

Similar results as in TIME were obtained in BedMed and BedMed-Frail trials. Again, patients were randomized to take their usual o.d. antihypertensive medication either in the morning or in the evening. Neither in 3357 adults participating in BedMed and followed over 4.6 years, nor in 776 residents of continuing care wards of BedMed-Frail followed over 415 days significant differences were observed in the primary outcome of MACE or in secondary efficacy and safety outcomes. More details can be found in different publications on study protocol [174] and results [175,176]. Recent evidence on so-called chronotherapy issues thus indicates that bedtime administration of antihypertensive drugs should not be recommended in routine practice to improve cardiovascular outcomes, and that the primary goal should be 24-h BP control using long-acting drugs in a single morning pill [170]. Further research is needed, however, to clarify whether more individualized approaches, e.g. evening administration of shorter acting drugs in selected patients with confirmed inadequate BP control at night, may be beneficial. Consideration of patient adherence and individual drug characteristics remains crucial in tailoring antihypertensive treatment strategies (Box 3).

Box 3 **Problems with chronotherapy-based evening drug dosing in hypertension**

**Table:** 

- Little pharmacologic rationale when long-acting drugs are used - Questionable efficacy in terms of nocturnal BP reduction - No evidence of reduction in medication-related adverse events - No outcome benefits in recent trials - May lead to less frequent use of recommended single pill combinations - Worse treatment adherence - Class specific adverse effects (e.g., nocturia with diuretics)

### Renal denervation

There is clear evidence of sympathetic overdrive in various clinical conditions including resistant hypertension associated with nocturnal BP elevation, as assessed by muscle sympathetic nerve activity recordings [177]. The rationale for renal denervation as a therapeutic intervention is based on removing the sympathetic nervous overdrive, specifically at the level of renal nerves and renal efferent sympathetic outflow. This intervention aims to disrupt the stimulation of the kidney which leads to vasoconstriction, renin secretion, hyperaldosteronism, and sodium retention, resulting in hypertension often resistant to treatment.

Experimental evidence in spontaneously hypertensive rats shows that renal denervation determines significant improvement in BP control, renal catecholamine levels and markers of renal and cardiac interstitial fibrosis [178].

The effectiveness of renal denervation in humans is demonstrated in trials such as the RADIANCE-HTN SOLO study [179] where endovascular ultrasound renal denervation reduced ambulatory BP at 2 months in patients with combined systolic-diastolic hypertension in the absence of medications. Similar evidence was seen in the SPYRAL HTN-OFF MED Pivotal study where radiofrequency renal denervation was superior compared with a sham procedure to safely lower ambulatory BP in the absence of antihypertensive medications [180]. Lastly, even on top of triple antihypertensive therapy, renal denervation was shown to be effective in reducing 24 h BP [181]. Pooled analysis of these three trials confirms a significant drop in ambulatory systolic BP, with a specific impact on night-time BP of on average 8 mmHg [182]. A recent analysis of a subset of SPYRAL HTN-ON MED participants with true resistant hypertension suggested that renal denervation might be particularly effective in controlling nocturnal BP in this group [183].

Renal denervation has shown promise in conditions such as obstructive sleep apnoea, where sympathetic nervous system activity is known to be heightened: in a randomized controlled trial, renal denervation lowered both office and ambulatory BP in patients with resistant hypertension and OSA and this was accompanied by improvement of the clinical severity of OSA [184].

Night-time BP appears to be a predictor of the response to renal denervation, with increased night-time BP levels and increased night-time BP variability prior to renal denervation associated with larger BP drops [185]. Additional factors, including heart rate and orthostatic hypertension, are also linked to better responses to renal denervation.

In conclusion, renal denervation effectively reduces night-time BP. This intervention holds potential benefits for patients with conditions such as obstructive sleep apnoea, and resistant hypertension [183], and mild CKD (eGFR between 45 and 60 ml/min/1.73 m2). Night-time BP, along with its variability, serves as a valuable marker for predicting responses to renal denervation.

### Improving sleep quality and duration

The Nobel Prize in Physiology and Medicine in 2017 highlighted the significant contributions of scientists in understanding molecular mechanisms controlling the circadian rhythm, which are also relevant to the study of human sleep. Sleep is a universal phenomenon with essential functions, including biological regulation, energy conservation, and cognitive functions like memory consolidation [186]. The amount of sleep needed varies with age, and the decline in sleep duration over the last 50 years is associated with lifestyle changes, increased use of technology, and elevated work burden, often requiring shift work.

As mentioned above, short-duration sleep sustained over time is linked to various health risks, including obesity, hypertension, diabetes, and cardiovascular diseases [187–191]. Conversely, sleep extension by 1 h per day can improve BP profile as seen in a randomized controlled study on healthy adults [19].

Poor quality of sleep, particularly factors like difficulty in falling asleep and insomnia [192] but also fragmented sleep with elevated arousal burden [193], is also associated with increased cardiovascular risk. Lifestyle and environmental changes, are recommended to improve sleep, along with addressing underlying conditions like sleep apnoea. Sleeping pills do not seem to have an impact on mortality hazards according to a retrospective cohort study on 34,727 patients followed-up for a mean of 7.6 years [194] However, the combination of hypnotics and extremely short or long sleep duration can further increase mortality risk as shown in a cohort of 484916 adults in Taiwan [195].

Shift work and circadian misalignment can disrupt sleep and contribute to health issues [196]. Occupational strategies, such as limiting shift duration and creating conducive environments, can help mitigate the negative impact of shift work. Importantly, creating an enabling environment at societal levels, addressing factors like noise, light exposure, air quality and food security is crucial for promoting overall sleep health [186].

### Treating sleep apnoea to reduce nocturnal BP

Sleep apnoea is characterized by repeated episodes of disrupted breathing during the night, often due to the narrowing of the upper airway. As previously discussed, a clear association between sleep apnoea and incident and prevalent hypertension was consistently demonstrated, with elevated prevalence of nondipping among OSA patients. It was thus proposed that OSA might be considered a cause of secondary hypertension, its treatment potentially improving BP control.

Interventions for sleep apnoea mainly include body weight reduction, continuous positive airway pressure (CPAP), and the use of mandibular advancement device (MAD). Meta-analyses indicate a modest yet clinically significant reduction in BP with both CPAP and MAD use, particularly in patients with resistant hypertension [197,198]. This benefit was more pronounced for nocturnal BP compared to office BP in patients treated with CPAP [199].

The effect of CPAP treatment may thus be considered a possible addition to conventional antihypertensive treatment but cannot be expected to replace it in OSA patients. In fact, in the VALSAS trial, valsartan, an angiotensin receptor blocker, induced a fourfold higher decrease in mean 24-h BP than CPAP in untreated hypertensive patients with OSA [200]. Interestingly, whilst CPAP was associated with a limited nocturnal BP reduction, valsartan allowed a more pronounced and clinically significant nocturnal BP reduction (−1.3 ± 4.6 mmHg vs. − 7.4 ± 8.4 mm, *P* < 0.05), the best result being achieved by combining CPAP with pharmacological treatment.

As anticipated, MAD can also reduce BP in patients with OSA: a recent noninferiority trial [201], demonstrated that in 220 participants with moderate-to-severe OSA, hypertension and increased CV risk randomized to CPAP or MAD, the latter was noninferior to CPAP for reducing 24-h mean arterial BP. Most importantly, the between-group difference in effectiveness favoured MAD and was more pronounced for asleep than for awake BP.

Besides CPAP and MAD, lifestyle modifications, including weight management and improved sleep hygiene, play a crucial role in comprehensive treatment strategies [202].

Overall, the interventions aiming to control nocturnal BP in OSA are not mutually exclusive, and a combination of CPAP, MAD, antihypertensive drugs, and lifestyle changes should be employed to achieve optimal outcomes. According to a very recent individual patient data meta-analysis, the BP reduction induced by CPAP in OSA seems to be mostly a function of the initial BP levels [203].

## CONCLUSION

### Nocturnal BP in recent guidelines

The 2023 European Society of Hypertension (ESH) guidelines dedicate an entire section to night-time hypertension, encompassing various types of BP behaviour at night, specifically addressing epidemiology, diagnosis, and treatment under the heading “Specific Hypertension Phenotypes” (Section 14.8). Notably, such an emphasis on nocturnal aspects is unprecedented in the history of these guidelines.

These guidelines underscore the importance of accurate measurements, advocating for ABPM as the preferred method, given its consolidated ability to accurately assess night-time BP [77]. In this perspective, at variance from traditional practices, the guideline recommends maintaining consistency in the frequency of measurements throughout the day and night, suggesting three measurements per hour to ensure data quality and avoid the risk of insufficient information during critical nocturnal hours. Still, the methodological limitations of ABPM need to be considered to accurately evaluate and interpret asleep BP measurement [51].

To support this approach, historical research [204] emphasizes the limitations of intermittent BP measurements and their potential impact on accuracy. Acknowledging the challenges posed by the poor reproducibility of night-time BP patterns, the guideline advocates for repeated ABPM to achieve a more reliable assessment.

The 2023 ESH guidelines address the clinical importance of isolated nocturnal hypertension, as an example of masked hypertension phenotype, offering concise insights into its definition, prevalence, and demographic variations. Additionally, they explore the adverse effects on organ damage and outcomes associated with different nocturnal BP patterns.

Reproducibility concerns are echoed throughout, drawing attention to the dynamic nature of night-time BP patterns over time [102]. The 2023 ESH guideline indeed recommends multiple recordings to enhance pattern reliability, though acknowledging the practical challenges, particularly in untreated patients.

Regarding treatment approaches, the ESH guideline addresses various strategies for managing nocturnal hypertension but cautions about the limited scientific evidence supporting these interventions. Notably, this guideline highlights the lack of conclusive data on the impact of modifying nocturnal BP patterns on patient outcomes. Also on the background of the TIME study [172], which showed no significant differences in cardiovascular outcomes between morning and evening drug administration, this guideline notes potential areas for future research and emphasizes the need for individualized patient choices, especially when multiple medications are involved.

In summary, the 2023 ESH guidelines prioritize night-time BP assessment, advocate for 24 h ambulatory BP monitoring, and underscore the challenges in reproducibility and treatment decisions. The overarching theme is a call for a nuanced and individualized approach to nocturnal hypertension management, considering the current gaps in scientific understanding.

Of note, also the 2024 ESC Guidelines on management of elevated BP and hypertension dedicate a paragraph to this issue, discussing the prognostic relevance of nocturnal hypertension and treatment-related aspects, while emphasizing the limited reproducibility of dipping patterns [205].

### Knowledge gaps

In 1988, Eoin O’Brien highlighted the higher risk associated with individuals with reduced or absent nocturnal BP reduction whom he termed nondippers [92]. After 37 years, the excess risk associated with an increased nocturnal BP has been confirmed, but the optimal management of these individuals remains uncertain. Current guidelines acknowledge the importance of examining such patients, as well as the uncertainty regarding the appropriate actions. Recognizing the variability in individuals’ behaviour, it is emphasized that a single 24 h ABPM to evaluate nocturnal BP may not suffice. Repeat measurements, potentially utilizing new techniques based on home nocturnal BP monitoring, might be useful because of the evidence suggesting their better accuracy and repeatability. The call for additional research is emphasized to better understand the potential applications of nocturnal home monitoring and to gather more data on this approach. Also, the possible role of cuffless BP monitoring devices is promising and requires further investigation in this field.

### Key issues for future research

- *Night-time blood pressure significance*: more data is needed to support the crucial role of night-time BP as a superior predictor of cardiovascular events, surpassing the significance of daytime BP, while accounting for unmeasured confounding.
- *Consistency in measurement frequency during 24 h ABPM*: Available guidelines advocate for maintaining consistency in BP measurement frequency throughout the day and night, emphasizing the importance of three measurements per hour to ensure data quality and prevent insufficient information during critical nocturnal hours. Further data on this issue is needed, also on the background of the introduction in clinical practice of home BPM devices equipped with nocturnal BP measurement function, currently providing only 3 BP measurements per night
- *Need to identify the optimal method to define wake and sleep time periods*: Night-time BP does not always correspond to actual sleep BP, and nocturnal sleep BP might differ to sleep BP during daytime naps. The method used to identify the wake and sleep time periods over 24 h leads to sleep BP quantification which varies according to whether it is based on narrow or wide fixed time intervals, on the objective estimation of time in bed through actigraphy or on actual sleep time definition by polysomnography with EEG recording. How such different definitions of sleep BP can affect dipping patterns, dipping classification and its prognostic value needs further investigation.
- *The possibility to improve nocturnal BP assessment by adoption of emerging technologies estimating BP by means of cuffless devices* requires further investigation. This would require the implementation of ad-hoc validation protocols able to test the ability of these devices not only to accurately estimate BP values, but also to reliably track their changes over time, including BP fluctuations between day and night.
- *Need to address reproducibility concerns related to assessment of day-night BP changes.* Current guidelines acknowledge the poor reproducibility of day-night BP patterns, recommending repeated 24 h ambulatory BP monitoring to achieve a more reliable assessment over time. More specific recommendations, also related to the possible usefulness of a better standardization of behaviours during 24 h ABPM are needed.
- *Need to better define the relevance and the management of isolated nocturnal hypertension,* a specific type of masked hypertension. More research is needed on its optimal definition, its prevalence and demographic variations, its impact on organ damage and outcome, and its management.
- *Need to define best treatment strategies for nocturnal BP elevation and to demonstrate the impact on outcome of its control.* Current guidelines caution about the limited scientific evidence on the impact of nocturnal BP patterns modulation on patient's outcomes. Further studies are needed to better address the actual benefit of “chronotherapy” implementation in clinical practice. Consideration should be given to a number of as yet unmeasured patient's characteristics (e.g. sleep quality and duration, individual chronotype, impact of shift-work) to be included in the baseline assessment of hypertensive patients, to improve risk assessment, and to provide appropriate circadian alignment of antihypertensive drug dosing time, aimed to maximize benefits.
- *Call for nuanced and individualized approach*: In summary, the overarching theme is a call for a nuanced and individualized approach to the management of nocturnal hypertension, considering the current gaps in scientific understanding.

## TAKE HOME MESSAGGES FOR DAILY PRACTICE, BASED ON CURRENT EVIDENCE

### Mechanisms of day-night BP changes, relevant for clinical practice

1. Changes in BP and heart rate between sleep and wakefulness are largely due to modulation of the autonomic nervous system, with an elevated sympathetic nervous activity being linked to nocturnal hypertension and a nondipping BP profile
2. The interaction of circadian rhythms, sleep architecture, and sleep microstructure plays a crucial role in night-time BP variations. There is evidence for a circadian rhythm in renin, aldosterone, cortisol and vasopressin release
3. Hypoxia, including intermittent hypoxia in OSA, leads to increased night-time BP, with a prominent role played by the arterial chemoreflex.
4. There is a bidirectional relationship between renal function and nocturnal BP. Poor sodium excretion and reduced kidney function, with intrarenal RAS activation during night-time, may contribute to elevated night-time BP and nondipping pattern

### Indications on how to perform nocturnal BP measurement in clinical practice

1. Assessment of nocturnal BP in clinical practice should currently rely on intermittent 24-h brachial cuff-based ABPM.
2. Given however the important practical limitations of this approach, new techniques in this field are being developed and investigated, including HBPM devices with nocturnal BP measurement function, which are currently being evaluated also for their easier applicability to repeated nocturnal BP evaluation.
3. A number of wearable devices are being produced and suggested to improve nocturnal BP assessment due (in case of cuffless devices) to avoidance of interference with sleep quality. However, solid and unbiased clinical evidence on the accuracy of these devices is still lacking making it impossible to recommend them for use in clinical practice at present.
4. Regarding cuffless BPM devices, there is need for independent clinical validation studies based on internationally accepted protocols testing their ability not only to accurately estimate BP values at rest, but also to track their changes over time.
5. Accurate definition of sleep time, and use of accurate and minimally intrusive BPM methods might help optimizing the definition of BP dipping patterns at night.

### How to define wake and sleep periods

1. Different methods have been proposed to identify daytime (awake) and night-time (sleep) periods. Among them, an acceptable compromise between practical applicability and accuracy is the adoption of diary entries best if accompanied by actigraphy recording of daily activities.
2. In absence of these tools, adoption of narrow-fixed time intervals may allow for identification of wake and sleep time periods during 24 h more precisely than by relying on wide-fixed time intervals.

### Importance of nocturnal BP assessment in relation with outcome

Assessing nocturnal BP is important because of its major impact on cardiovascular outcome, including stroke, in treated hypertensive individuals. Its accurate and repeated assessment may contribute to better and smoother 24 h BP control and to cardiovascular (CV) risk reduction.

### Dipping vs. nocturnal hypertension

1. Both average night-time BP levels and the day-night BP dip are robust and partly independent predictors of cardiovascular risk, although nocturnal BP level appears to be superior in this regard.
2. The clinical utility of BP dip classification remains uncertain, also due to its low reproducibility, especially in patients reporting significantly disturbed sleep during monitoring.
3. Therefore, the clinical interpretation of nondipping should take into account sleep quality and repeated ABPM should be considered to confirm abnormal nocturnal BP patterns.

### Special populations in which nocturnal BP assessment is indicated because of high prevalence of nocturnal BP alterations

1. Neurological disorders associated with autonomic neuropathy.
2. Sleep disorders characterized by alterations in sleep duration and quality, including sleep disordered breathing.
3. Kidney disorders.
4. Metabolic and endocrine disorders, including overweight and obesity, diabetes and metabolic syndrome, increased aldosterone secretion, Cushing's syndrome, pheocromocytoma.

### Therapeutic aspects

1. Controversial evidence on usefulness of chronotherapy.
2. Available hypertension management guidelines do not recommend systematic evening antihypertensive drug administration, unless there is a specific need to focus on nocturnal BP.
3. Use of long-lasting antihypertensive drugs, better if in combination, administered at a time which guarantees the best adherence in individual patients is recommended.
4. Evening administration of short acting antihypertensive drugs may be useful in patients with isolated nocturnal BP elevation, accompanied by daytime hypotension, associated with autonomic dysfunction.
5. Renal denervation reduces nocturnal hypertension, with night-time BP, along with its variability, representing a valuable marker for predicting responses to renal denervation.
6. Improvement of sleep quality and duration has a favourable impact on nocturnal BP and on outcome.
7. Treatment of sleep apnoea is associated with a reduction of an elevated nocturnal BP.

## ACKNOWLEDGEMENTS

This work has not been previously presented.

Sources of support/funding: none.

### Conflicts of interest

Conflicts of interest in relation to this paper: Krzysztof Narkiewicz: speaker honoraria from Omron and Medtronic; Gianfranco Parati: speaker honoraria from Omron and Somnomedics; George Stergiou: lecture and consulting fees by A&D, Huawei, Livemetric, Microlife, Omron, SkyLabs, Sonion. No reported conflicts of interest for the other authors.
